# Wfs1 and Related Molecules as Key Candidate Genes in the Hippocampus of Depression

**DOI:** 10.3389/fgene.2020.589370

**Published:** 2021-01-22

**Authors:** Jing Yang, Chaoqin Chen, Xiaoyuan Jin, Lu Liu, Jiajia Lin, Xianhui Kang, Shengmei Zhu

**Affiliations:** ^1^Department of Anesthesiology, The First Affiliated Hospital, Zhejiang University School of Medicine, Hangzhou, China; ^2^Department of Public Health, University of Minnesota Twin Cities, Minneapolis, MN, United States; ^3^Department of Neurobiology, The Innovation Center for Brain Science, Institute of Neuroscience, Zhejiang University School of Medicine, Hangzhou, China

**Keywords:** depression, Hippcampus, Wfs1, CUMS, microRNAs

## Abstract

**Background:**

Depression is a prevalent mental disorder, which is difficult to diagnose and treat due to its unclear pathogenic mechanisms. The discovery of novel and effective therapeutic targets for depression is urgently needed. The hippocampus is a crucial region involved in depression and has been a therapeutic target for many antidepressants. Thus, it is beneficial for comprehensive research to be carried out on the molecular mechanisms of the hippocampus involved in the pathogenesis of depression. This study aims to investigate the differentially expressed genes (DEG) in the hippocampus in a chronic unpredictable mild stress (CUMS) mouse model.

**Method:**

The study obtained GSE84183 from the GEO database. The R language screened the differential expression genes (DEG) in the hippocampus tissue of depressed mice, and the enrichment pathways of DEGs were analyzed. A protein-protein interaction (PPI) network was constructed in the STRING database and visualized in Cytoscape software. MicroRNAs for these DEGs were obtained from TarBase and mortar base databases, and transcription factors (TF) related to DEG were predicted from the ENCODE database. Both networks used the visual analysis platform NetworkAnalyst. Finally, the microRNA-TF network was integrated based on the above two networks and imported into Cytoscape for further analysis.

**Results:**

This study screened 325 differentially expressed genes, containing 42 downregulated genes and 283 upregulated genes. Most of these genes are enriched in the cell cycle and the chemokine signaling pathway. Meanwhile, Wfs1, one of the top ten DEGs, was identified as the key regulator of the cell cycle and the participator in the highest number of modules screened out in PPI networks. Wfs1-related molecules, including UBTF, mmu-mir-17-5p, and mmu-mir-7b-5p, were therefore screened out. Furthermore, we confirmed the downregulation of Wfs1 and upregulation of UBTF/mmu-mir-17-5p/mmu-mir-7b-5p in the hippocampus of the CUMS mouse model. Our data indicate that Wfs1 and related molecules were predicted to be associated with the pathological process of depression. This research provided potential new molecular targets of stress-induced depression.

## Introduction

Depression is one of the most common and devastating neuropsychiatric diseases, and has become a severe health problem worldwide (Etkin et al., 2015). Studies have shown that about one-third of patients with depression are unresponsive to conventional antidepressant therapy (Rush et al., 2006). The development of new biomarkers for the treatment of depression is urgently required. A more in-depth exploration of the molecular mechanisms involved in the pathological process of depression may reveal new ways helps to combat this complex disease.

Depression is associated with smaller hippocampal volumes and changes in either the activation or connectivity of neural networks (Etkin et al., 2015; Ma et al., 2019). Depression also increases the level of inflammatory factors in the hippocampus (Fasick et al., 2015), downregulates BDNF (Krishnan and Nestler, 2008), and impairs synaptic plasticity (Otte et al., 2016), all of which contribute to the development and long-term pathophysiology of depression. These studies show that hippocampus dysfunction occurred in the neuropathology of depression.

Microarray technology can quickly screen out differential gene expression information related to depression. According to bioinformatics analysis, the functional pathway of differentially expressed genes (DEG) in the pathogenesis of depression can be analyzed. Herve et al. (2017) screened the gene expressions related to depressive behavior after chronic stress in the GSE84183 data set in 2017. It is worth noting that that study only paid attention to and studied the function of a select few of these genes. In order to obtain a more comprehensive understanding of the pathogenesis of depression, we used a variety of bioinformatics techniques to reanalyze some of the data in the GSE84183 data set. This study constructed a TF-DEG network and a microRNA-DEG network based on the DEG identified from CUMS samples and standard control samples. The regulatory association between TF and its target is obtained from the “Encyclopedia of DNA Elements” (ENCODE). The association between microRNA and its target is extracted from the TarBase and mortar base databases. These were then integrated into the network and the TF-microRNA core regulatory network was built, from which key microRNAs (mmu-mir-17-5p and mmu-mir-7b-5p) and TF (UBTF) in depression can be identified. The present study highlights and identifies Wfs1 and related molecules in the hippocampus as new potential targets for depression and improves our understanding of molecular mechanisms underlying depression pathophysiology.

## Materials and Methods

### Microarray Data Data Set Collection and Identification of DEGs

The study obtained microarray expression data from the Gene Expression Omnibus^1^. Based on the Agilent GPL13912 platform (Agilent-028005 SurePrint G3 Mouse GE 8 × 60K Microarray), the exon expression profiling provided eight sham and eight CUMS hippocampus. The probes were converted to the corresponding gene symbols according to the annotation information of the raw data. To reduce multiple testing, each corresponded to a unique gene symbol. Only the probe set with the highest average expression was considered when multiple probe sets were associated with the same gene. The pre-treatment standardization on gene expression data for each experiment was performed using the R/Bioconductor Limma package. After the linear model fitting, the Bayesian linear model of the Limma package was estimated to identify DEGs. Statistically significant DEGs were defined with *P* < 0.05 and | logFC| > 1 as a cut-off criterion in this study. Heatmap and volcano plot visualizations were performed using the R package “pheatmap” and “ggplot2,” respectively.

### Enrichment Analyses of DEGs

In this study, gene ontology (GO) enrichment and Kyoto Encyclopedia of Genes and Genomes (KEGG) pathway analysis of DEGs was carried out using R. We also performed gene set enrichment analysis (GSEA) using the “clusterProfiler” package in R (Yu et al., 2012). All visualization was handled in R using the ggplot2 graphics package.

Metascape is a powerful enrichment analysis online tool^2^, which can display the activation levels of biological pathways. Because the database is often updated, it has high credibility and accuracy, which helps bioinformatics analysis (Zhou et al., 2019).

### Module Screening From the PPI Network

The comprehensive information of the proteins found by using STRING^3^, a search tool for retrieving interacting genes/proteins, was used to evaluate protein-PPI information. Subsequently, a PPI network was constructed and visualized by Cytoscape software (version 3.7.1). Then, Molecular Complex Detection (MCODE) analysis, an APP of Cytoscape, was used to select the most significant PPI network modules. The criteria for selection were as follows: MCODE score > 3, degree cut-off = 2, node score cut-off = 0.2, and max depth = 100.

### TF-DEG Network and MicroRNA-DEG Network Construction

NetworkAnalyst^4^ is a comprehensive web-based tool for network-based visual analytics of comprehensive gene expression profiling and meta-analysis. All the identified DEGs were uploaded to NetworkAnalyst to obtain TF-gene and microRNA-gene interaction data. The generated list of datasets was exported to Cytoscape for further analysis.

### TF-MicroRNA Synergistic Regulatory Network

The TF–miRNA regulatory network was built by integrating TF-target and miRNA-target, and the TF-target-miRNA network was constructed using Cytoscape. The DERs that showed reciprocal changes in TF and microRNA were selected, and the microRNAs and TFs associated with them were extracted. Finally, the network was obtained and visualized in Cytoscape.

### Animals and CUMS (Chronic Mild Unpredictable Stress, CMUS) Models

Adult male ICR mice (20–25 g) purchased from the Experimental Animal Center of Zhejiang Province (Hangzhou, China) were used in this study. The animals were housed in a temperature-controlled animal facility with a 12 h light-dark cycle. All procedures were approved by the Animal Care and Use Committee of Zhejiang University, following the guidelines for the Care and Use of Laboratory Animals by the National Institute of Health (NIH Publications No. 80-23).

Chronic unpredictable mild stress is a mouse model of depression in which animals are exposed to a random sequence of mild stressors and make a slight modification based on there already had studies (Jiang et al., 2019). Mice were subjected to 10 different stressors: tail pinching (3 min), electric shock to the sole (voltage 30 mV, 10 s/time, 1 min interval, 30 times in total), water deprivation (24 h), food deprivation (24 h), light-dark cycle reversal, hot environment (45°C, 5 min), swimming in cold water (4°C, 5 min), cage shake (15 min), wet bedding (24 h), cage tilt (24 h), and restraint (8 h). These stressors were performed every day in a random sequence.

### Depression Behavioral Tests

#### Forced Swimming Test (FST)

The test was performed as previously described. Mice were individually placed into a glass cylinder (15 cm diameter, 30 cm height). The water depth was around 20 cm to prevent mice from touching the cylinder bottom with tails or limbs. The water temperature was maintained at 23–25°C. Black cardboard was placed between every two cylinders to minimize the interaction of mice. Each mouse was allowed to swim for 6 min. A digital camera videotaped all test sessions. The immobility time during the last 4 min was scored offline. Immobility time was defined as when the mice were motionless or passively floating on the water (only with movements necessary for keeping balance).

#### Sucrose Preference Test (SPT)

Mice were singly housed and habituated with one sucrose bottle (1%, w/v) and one bottle of water for 48 h (from day-2 to day-1). Bottle positions were switched after 24 h, and mice were then immediately water-deprived for 24 h. During the test session, mice were singly housed and exposed to one bottle of 1% sucrose and one bottle of water for 24 h. Bottle positions were switched after 12 h. Total consumption of each fluid was measured, and the sucrose preference was calculated according to the following ratio: sucrose preference (%) = [sucrose intake (g)/sucrose intake (g) + water intake (g)] × 100%.

### Real-Time Polymerase Chain Reaction (RT-PCR)

The RT-PCR analysis was performed as previously described (Yang et al., 2019). Four weeks after CUMS, our control mice were deeply anesthetized with an overdose of sodium pentobarbital (150 mg/kg i.p.) and sacrificed. Freshly isolated hippocampus tissues were collected on ice, immersed in TRIzol reagent (Invitrogen, Carlsbad, CA, United States), and immediately stored at −80°C until the time of RNA extraction. Complementary DNA (cDNA) synthesis was synthesized with the reverse transcription enzyme SuperScript II (Invitrogen, Carlsbad, CA, United States) together with the reverse transcription primer. The cDNA was then amplified using HiFiScript cDNA Synthesis Kit (CWBIO, Beijing, China) by ABI Q5 RT-PCR System (Applied Biosystems, Thermo Fisher, United States). All primers were synthesized by BGI Co., Ltd. (Shenzhen, China) (Table 1).

**TABLE 1:** Primer used in this study.

Gene	Primers	Sequence
GAPDH	Forward primers	AAGGTCGGTGTGAACGGATT
	Reverse primers	TGAACTTGCCGTGGGTAGAG
Wfs1	Forward primers	GGAAACTAACATGGCCCGGA
	Reverse primers	GTGATGGAGGGTCTTTGGGG
UBTF	Forward primers	ACAACCTCCCATCCAACGAC
	Reverse primers	TGTTGTGTTGACCCCTCCAC
U6	Forward primers	AGAGAAGATTAGCATGGCCCCTG
	Reverse primers	ATCCAGTGCAGGGTCCGAGG
mmu-mir-17-5p	Forward primers	ACACTCCAGCTGGGCAAAGTGCTTACAGTGCAG
	Reverse primers	CTCAACTGGTGTCGTGGA
mmu-mir-7b-5p	Forward primers	TCCAAGACATGTGATCCGTGTT
	Reverse primers	GGGTTACTCCCGTTTGGTTGT

### Data and Statistical Analysis

Data are presented as means ± SE. Analyses were performed using Microsoft Excel. Students’ *t*-test tests were used for analyzing statistic differences, as indicated in Figure legends. *p* < 0.05 was considered as statistically significant.

## Results

### Data Normalization

**FIGURE 1:**
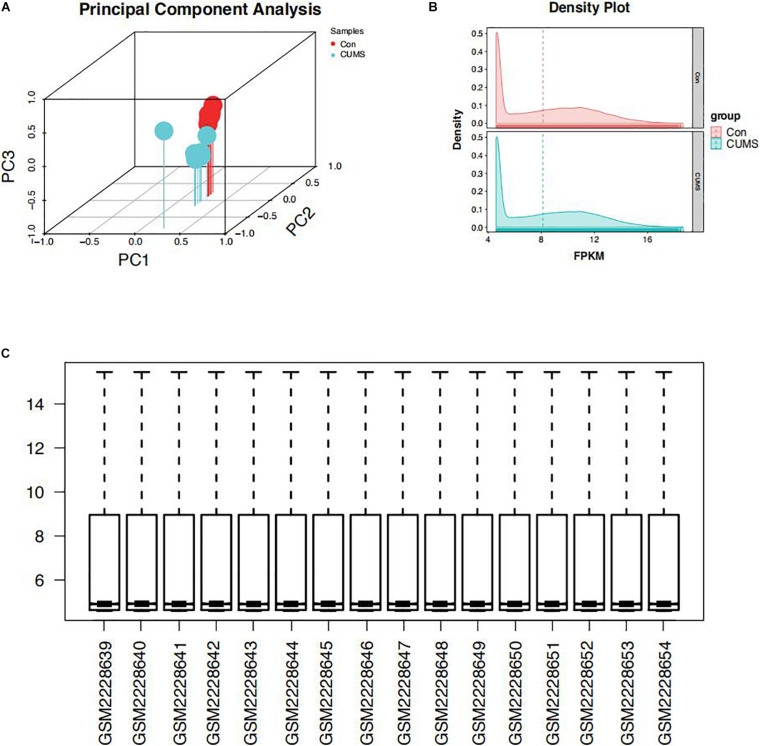
Distribution features of the expression situation after normalization of all samples. **(A)** Principal component analysis (PCA) graph. **(B)** The density plots. **(C)** A box plot.

The primary purpose of normalization is to eliminate technical and systematic variability from the data to compare different samples. After microarray data normalization, biological variability between different samples was assessed by plotting a principal component analysis (PCA) graph (Figure 1A). As can be noted, CUMS (*n* = 8) and Control (*n* = 8) overall had distinct, non-overlapping expression profiles. The density plots results demonstrated that the distributions of the samples’ intensities were generally consistent and could be used for downstream analysis (Figure 1B). A box plot showed each sample’s gene expression, and the black lines in the boxes were almost on the same straight line, indicating that the raw data were normalized successfully, which ensures the accuracy of the data (Figure 1C).

### Identification of DEGs

**FIGURE 2:**
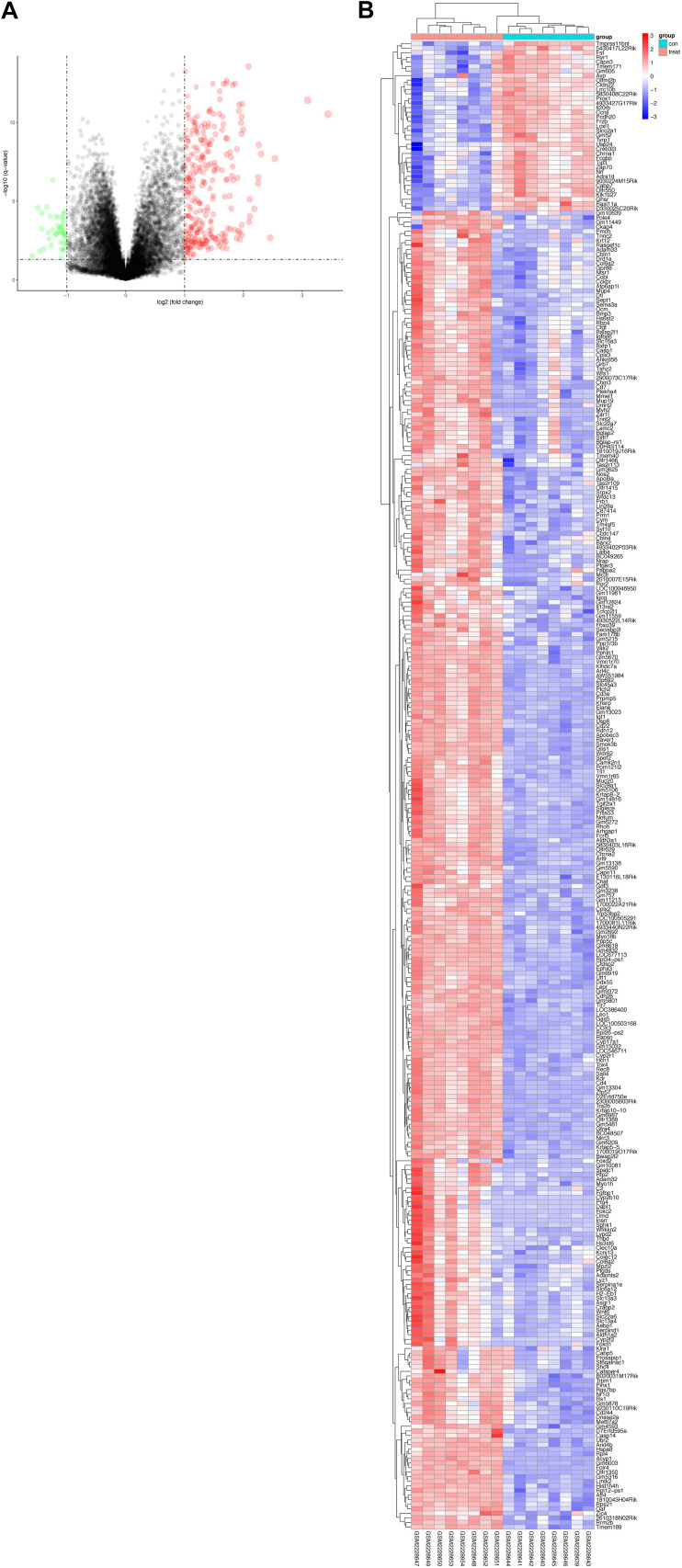
The volcano plot **(A)** and clustering map **(B)** were generated using the R package “ggplot2.” **(A)** Volcano Plot indicated upregulated and downregulated mRNAs of the hippocampus in CUMS model. Red represents the selected upregulated genes and green represents the selected downregulated genes. **(B)** RNA-seq comparing hippocampus from mice exposed to CUMS or control group. Eight biological replicates were used per condition for the hippocampus. Hierarchical cluster heatmaps of the hippocampus showing relative expression of genes across sham and CUMS samples.

Based on the Limma package in R language, *P* < 0.01 and | logFC| > 0.5. A total of 325 DEGs were screened, including 283 upregulated and 42 downregulated DEGs. The volcano of DEGs is presented in Figure 2A and heatmaps plots in Figure 2B.

### Functional Enrichment Analysis of DEGs

**FIGURE 3:**
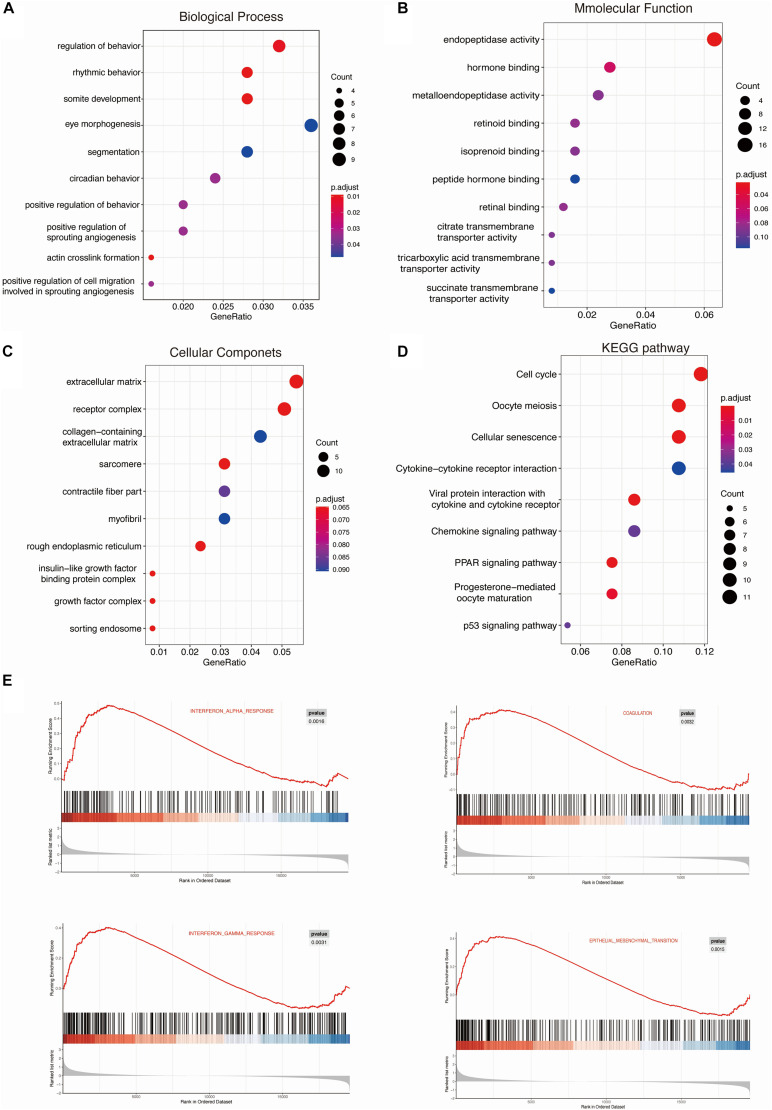
KEGG and GSEA enrichment analysis of the DEGs were performed using ClusterProfiler. Gene ontology (GO) analysis for CUMS models DEGs: Biological Processes (BP), Molecular Function (MF), and Cellular Components (CC) **(A–C)**. **(D)** The Kyoto Encyclopedia of Genes and Genomes (KEGG) pathways enrichment of DEGs. **(E)** h.all.v 6.2.symbols.gmt [Hallmarks] gene set database was used to analyze the whole gene expression value of the PTB and HC samples. Significant gene sets were cut-off by FDR < 0.25 and *P*-value < 0.05.

To further investigate the function of DEGs, GO term, and KEGG pathway analyses were displayed in R. These DEGs were all divided into three major functional categories: biological processes (BP), molecular functions (MF), and cellular components (CC). The data collectively showed that DEGs were largely involved in circadian rhythm, cytokine biosynthesis processes, and prostaglandin biosynthesis processes (Figure 3A and Table 2). As for the molecular function (MF) group, DEGs were mainly enriched in endopeptidase activity (Figure 3B). In the CC analysis, the DEGs were predominantly enriched in the extracellular matrix receptor complex (Figure 3C). In these candidate DEGs, 14 signaling pathways were enriched in the KEGG database, such as Cell cycle, Oocyte meiosis, and Cellular senescence (Figure 3D).

**TABLE 2:** Top 10 biological process of DEGs.

Term	Genes	*P* value
GO: 0050795 ∼ circadian rhythm	Lepr/Ptger3/Mc3r/Ghsr/Pmch/Ptgds/Casp1/Cckbr	9.60E-06
GO: 0007622 ∼ cytokine biosynthesis processes	Lepr/Chat/Ptger3/Mc3r/Pmch/Ptgds/Casp1	5.17E-06
GO: 0061053 ∼ prostaglandin biosynthesis processes	Gdf3/Frzb/Ror2/Foxc2/Dmrt2/Cobl/Aldh1a2	7.71E-05
GO: 0048592 ∼ synaptic vesicle fusion on the regulation of presynaptic active zone membrane	Kdr/Vax2/Hcn1/Trpm1/Rs1/Prox1/Rbp4/Nrl/Col8a2	0.000130269
GO:0030335∼segmentation	Gdf3/Sema3a/Ror2/Foxc2/Dmrt2/Cobl/Aldh1a2	7.88E-13
GO: 0048512 ∼circadian behavior	Lepr/Ptger3/Mc3r/Pmch/Ptgds/Casp1	5.02E-05
GO: 0048520 ∼positive regulation of behavior	Ptger3/Ghsr/Pmch/Casp1/Cckbr	6.79E-05
GO:0045087∼ positive regulation of sprouting angiogenesis	Kdr/Srpx2/Ghsr/Foxc2/Fgfbp1	2.01E-09
GO: 0051764 ∼actin crosslink formation	Baiap2l2/Tnnt2/Baiap2l1/Cobl	4.11E-06
GO: 0090050 positive regulation of cell migration involved in sprouting angiogenesis	Kdr/Srpx2/Foxc2/Fgfbp1	6.85E-05

Moreover, we used more than one kind of enrichment analysis to ensure the accuracy and reliability of the data analysis. Therefore, 325 differentially expressed genes were uploaded to the Metascape for standardized pathway and molecular function analysis. Biological processes (BP) and KEGG enrichment results show that DEGs were mainly enriched in rhythmic behavior, negative regulation of interleukin-1 beta production, and cell apoptotic pathways (Supplementary Figure 1). The enrichment results of meta scope are consistent with the R language. Also, we performed GSEA analysis for all genes on the microarray. The significantly enriched gene sets were set at a default cut-off as *P*-value < 0.05 and FDR < 0.25. Furthermore, the consequences of GSEA analysis suggested that the expression profiles of CUMS model were primarily enriched in “interferon-alpha response,” “coagulation,” “interferon-gamma response,” and “epithelial-mesenchymal transition” (Figure 3E).

### PPI Network and Module Analysis

**FIGURE 4:**
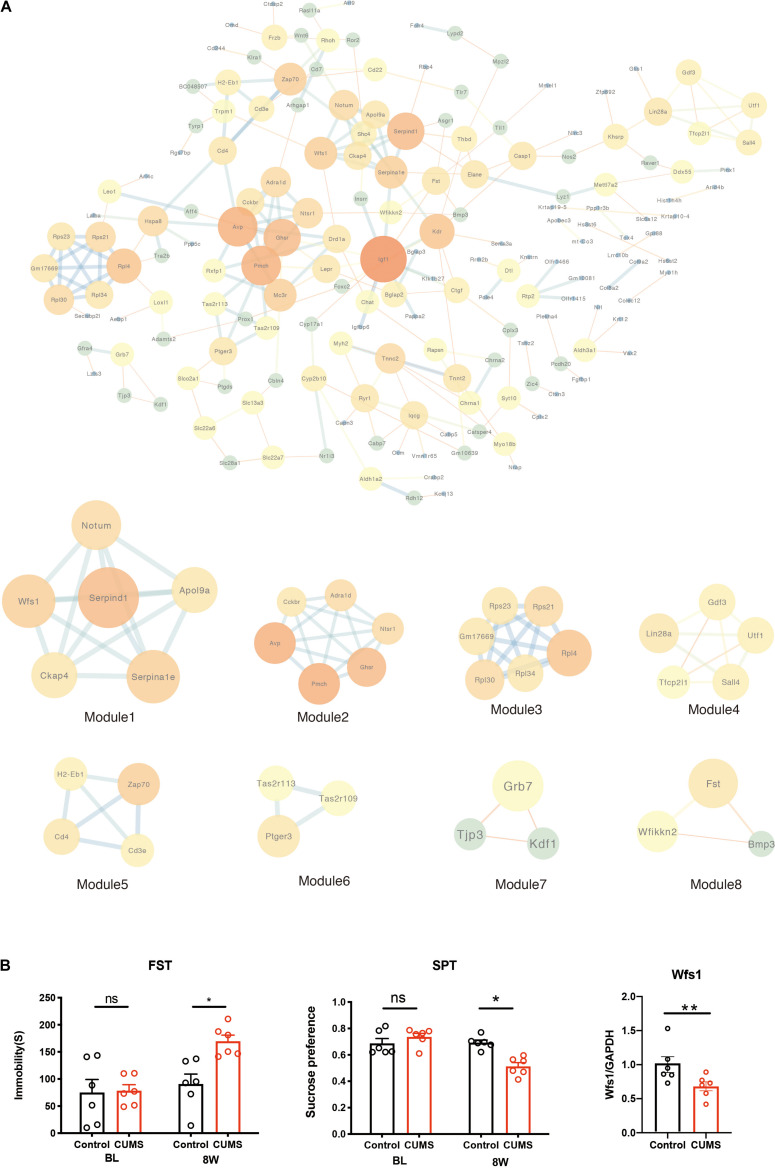
PPI network of DEGs constructed by the STRING database. **(A)** The PPI network of DEGs was constructed using Cytoscape. Identification of a sub-network using MCODE in Cytoscape software. **(B)** Depression behavior after chronic mild unpredictable stress. CUMS increased the immobility time compared with control mice, and decreased the sucrose preference compared with control mice. Real-time PCR verified the downregulation of hippocampal Wfs1 gene expression after CUMS. one symbol *p* < 0.05, two symbols *p* < 0.01 (*n* = 6 mice/group).

To investigate the relationship between protein interaction by those DEGs, the PPI network was constructed in a STRING (V11.0), which consisted of 245 edges and 174 nodes. The STRING analysis showed that a total of 174 genes were filtered into the DEGs PPI network complex. The network was visualized using the software tool Cytoscape (Figure 4A). Moreover, eight significant models were screened out from the PPI network according to the module analysis by MCODE in Cytoscape (Table 3). They screened out the modules with the highest MCODE analysis scores, including Serpind1, Ckap4, Wfs1, Notum, Serpina1e, and Apol9a (Figure 4A). Furthermore, we found that Wfs1 was overlapping within the top 10 DEGs (Cplx2, COX3, Ptgds, Hspa8, Rgs7bp, Raver1, Gm4832, Rpl4, Mettl7a2, and Wfs1). We compared the normal hippocampus on the 4 weeks with the treated group to verify the screened genes in real-time PCR. This was the most obvious time point for depressive behavior (Figure 4B). Consistent with our bioinformatics results, the expression of Wfs1 decreased significantly after stress (Figure 4B). Due to differences in the laboratory environment, experimental details, and different strains of mice, the final sacrifice time was determined by the depression phenotype of the mice. The asterisk indicates significant statistical difference (*P* < 0.05), four symbols *p* < 0.0001.

**TABLE 3:** Module analysis of DEGs using Cytoscape.

Cluster	Score	Nodes	Edges	Node IDs
1	6	6	15	Serpind1/Ckap4/Wfs1/Notum/Serpina1e/Apol9a
2	6	6	15	Ghsr/Ntsr1/Cckbr/Adra1d/Pmch/Avp
3	6	6	15	Gm17669/Rpl30/Rpl4/Rpl34/Rps21/Rps23
4	4.5	5	9	Gdf3/Lin28a/Sall4/Utf1/Tfcp2l1
5	4	4	6	H2-Eb1/Cd4/Cd3e/Zap70
6	3	3	3	Ptger3/Tas2r113/Tas2r109
7	3	3	3	Kdf1/Tjp3/Grb7
8	3	3	3	Bmp3/Wfikkn2/Fst
9	3	3	3	Rapsn/Chrna1/Chrna2

### Construction of the TF-DEG- MicroRNA Network Analysis

To further investigate the functional roles of DEGs, the potential regulatory relationships between DEGs and TFs were screened according to TF binding site data, and genetic coordinate position information provided on ENCODE (Supplementary Figure 2).

Next, we identified microRNA-DEG pairs through network analysis of 325 DEGs using the TarBase and miRTarBase databases, and five large-pairing pictures were generated (Supplementary Figure 3). As a result, a total of 436 associations between 257 microRNAs and only 71 DEGs were found.

**FIGURE 5:**
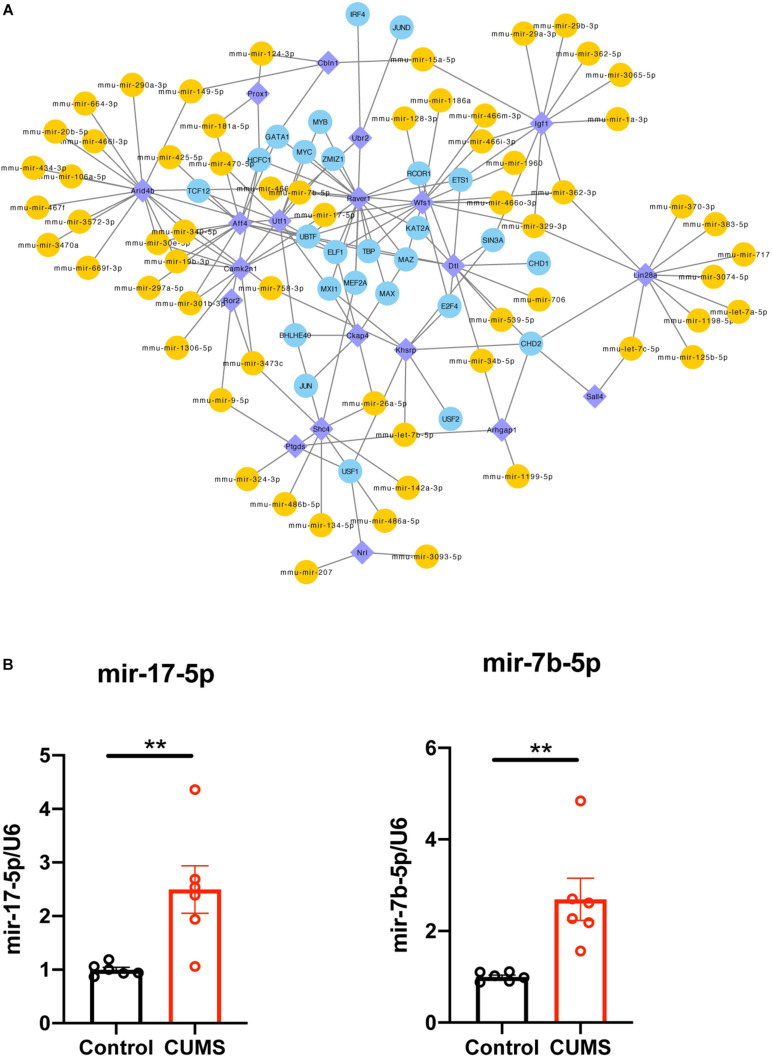
**(A)** Integrative regulatory network of TF-DEG-miRNA, DEG, differentially expressed gene; miRNA, microRNA; TF, transcription factor. **(B)** mRNA expression level of UBTF, mmu-mir-17-5p and mmu-mir-7b-5p were upregulated in hippocampus after CUMS group compared with control group. one symbol *p* < 0.05, two symbols *p* < 0.01 (*n* = 6 mice/group).

The co-regulated 20 DEGs modulated by microRNA and TFs were selected, and their related regulators were extracted, and then a TF-microRNA-interacted network in Cytoscape was constructed (Figure 5A). This network included 22 DEGs, 26 TFs, and 62 microRNAs, with 163 associations. We separately analyzed the degree of these 20 DEGs in the TF-DEG network and the microRNA-DEG network. Then, we found that Wfs1 regulated four TF (UBTF, ELF1, TBP, and MAZ) and directly regulated five miRNAs (mmu-mir-17-5p, mmu-mir-362-3p, mmu-mir-329-3p, mmu-mir-7b-5p, and mmu-mir-466i-3p) (Table 4).

**TABLE 4:** Co-DEGs regulated by TF and miRNAs.

TF	DEGs	Gene counts	miRNA	DEGs	Gene counts
UBTF	Arid4b, Aff4, Raver1, Wfs1, Dtl, Camk2n1	6	mmu-mir-17-5p	Wfs1, Camk2n1, Aff4	3
ELF1	Aff4, Utf1, Raver1, Wfs1, Ckap4	5	mmu-let-7b-5p	Ptgds, Khsrp, Arhgap1	3
CHD2	Arhgap1, Khsrp, Dtl, Lin28a, Sall4	5	mmu-mir-362-3p	Lin28a, Wfs1, Igf1	3
HCFC1	Prox1, Raver1, Aff4, Ror2, Cbln1	5	mmu-mir-329-3p	Lin28a, Wfs1, Igf1	3
TBP	Aff4, Raver1, Wfs1, Dtl	4	mmu-mir-3473c	Camk2n1, Ror2, Shc4	3
MXI1	Utf1, Raver1, Ckap4, Khsrp	4	mmu-mir-7b-5p	Camk2n1, Raver1, Ubr2	3
MAZ	Raver1, Wfs1, Ckap4	3	mmu-mir-19b-3p	Arid4b, Camk2n1, Aff4	3
E2F4	Wfs1, Dtl, Khsrp	3	mmu-mir-340-5p	Arid4b, Camk2n1, Aff4	3
BHLHE40	Ckap4, Utf1, Shc4	3	mmu-mir-30e-5p	Arid4b, Camk2n1, Aff4	3
ETS1	Igf1, Dtl, Raver1	3	mmu-mir-466f-3p	Wfs1, Arid4b	2
USF1	Khsrp, Ptgds, Nrl	3	mmu-mir-466i-3p	Wfs1, Igf1	2
SIN3A	E2F4, Igf1, Dtl	3	mmu-let-7c-5p	Sall4, Lin28a	2
TCF12	Aff4, TCF12	2	mmu-mir-124-3p	Cbln1, Prox1	2
MYC	Utf1, Raver1	2	mmu-mir-26a-5p	Shc4, Ckap4	2
MYB	Utf1, Raver1	2	mmu-mir-9-5p	Ptgds, Ror2	2
ZMIZ1	Utf1, Raver1	2	mmu-mir-181a-5p	Aff4, Prox1	2
MEF2A	Ubr2, Shc4	2	mmu-mir-758-3p	Khsrp, Arid4b	2
KAT2A	Dtl, Raver1	2	mmu-mir-15a-5p	Igf1, Cbln1	2
GATA1	Raver1, Aff4	2	mmu-mir-149-5p	Cbln1, Arid4b	2
RCOR1	Raver1, Dtl	2	mmu-mir-301b-3p	Camk2n1, Aff4	2

### Verification of Potential Experimental Target Expression by qRT-PCR

Four TF (UBTF, ELF1, TBP, and MAZ) and five miRNAs (mmu-mir-17-5p, mmu-mir-362-3p, mmu-mir-329-3p, mmu-mir-7b-5p, and mmu-mir-466i-3p) were verified, and mmu-mir-17-5p and mmu-mir-7b-5p were found to be highly reliable (cross-links ≥2) when targeting Wfs1. Then, using qRT-PCR analysis, selected molecules, including mmu-mir-17-5p, mmu-mir-7b-5p, and TF, were verified in the hippocampus (Figure 5B). Consistent with the prediction, the results showed that the expression levels of mmu-mir-17-5p, mmu-mir-7b-5p, and UBTF in the CUMS group were significantly higher than control mice. There was no significant difference in ELF1, TBP, and MAZ expression compared with the control group (Data not shown).

## Discussion

Depression is a complex neuropsychiatric disorder and alters multiple gene expression/signal cascades in the hippocampal region (Zandio et al., 2002). In this study, we screened 325 DEGs in the hippocampi of mice receiving chronic unpredictable stimulation. Based on a series of bioinformatics analyses, we identified Wfs1 and its related molecules as potential genetic targets for depression.

We screened out 325 differential genes and analyzed these DEGs through a variety of bioinformatics analysis methods. The results indicate that the GO terminology of 325 DEGs categorizes the depression-related biological processes as circadian rhythm, cytokine biosynthesis processes, and prostaglandin biosynthesis processes. It has been reported that the hippocampus regulates REM sleep disorders in depression. Chronic unpredictable stimulation will increase REM sleep time and downregulate the hippocampus and other brain regions (Lustberg and Reynolds, 2000; Zandio et al., 2002). A great amount of evidence supports the correlation between the upregulated expression of inflammatory cytokines in the hippocampus and depression (Makhija and Karunakaran, 2013; Song et al., 2018; Wang et al., 2018). KEGG pathway enrichment analysis shows that these DEGs are mainly related to the cell cycle, the interaction of cytokines and cytokine receptors, and the chemokine signaling pathways. The GSEA data is consistent with the above analysis results, and all genes are enriched in interferon-gamma alpha. Our findings suggest that the circadian rhythm, neuroinflammation, and cell cycle may play a vital role in the pathogenesis of depression.

Next, we constructed a PPI network to study DEG’s interaction relationship and screened out the modules with the highest scores in MCODE analysis, including Serpind1, Ckap4, Wfs1, Notum, Serpina1e, and Apol9a. These genes are highly related to depression. Consistently, Serpind1 is considered a candidate gene for depression (Wang et al., 2019). We noticed that Wfs1 is the overlapping gene of the first ten differential genes. The qRT-PCR results also show that the expression of Wfs1 in the CUMS group is downregulated compared to the control group. The above results indicate the importance of this gene in depression.

Wfs1 is an ER membrane protein and expressed specifically in the adult mouse brain regions relevant to stress and depression, including superficial layers of the cerebral cortex, the central extended area of the amygdala, and the hippocampus (Shrestha et al., 2015). Under external stimuli, Wfs1 knockout mice show stress responses. Locomotor activity of Wfs1-deficient mice was significantly lower only in a brightly lit environment. Short-term isolation had a significant anxiogenic-like effect on the behavior of Wfs1-deficient mice in a dark/light exploration test (Kato et al., 2008). To assess the regional specificity of Wfs1 loss in the mice, Shrestha et al. (2015) studied conditional knockout Wfs1 from forebrain neurons. Exposure of Wfs1 CKO (mPFC) mice to ARS (acute restraint stress) resulted specifically in enhanced responses to stress, including increased immobility in the FST and a suppressed preference for sucrose, and was accompanied by hyperactivation of the HPA axis and elevation of serum corticosterone (Shrestha et al., 2015). It should be noted that the depression model used in this study was ARS, an acute stress-induced depressive state. Here, we have reported the change of the Wfs1 mRNA level in the CUMS model, suggesting that Wfs1 is involved in the early state and the long-term development of depression. The present analysis supports a possible contribution of hippocampal Wfs1 to depression in the hippocampus is in its beginning stages. However, the detailed mechanism of Wfs1 in depression requires further investigations.

Transcription factors and microRNA have been proven to be important regulators in developing depression (Carlezon et al., 2005; Gururajan et al., 2016). Therefore, we constructed a TF-microRNA interaction regulatory network to predict the potential interaction of TF, microRNA, and DEGs in depression. UBTF is connected to the six target genes of the TF node (Arid4b, Aff4, Raver1, Wfs1, Dtl, and Camk2n1). Screening by qRT-PCR found that the expression of UBTF in the CUMS group was upregulated compared to the control group, suggesting a correlation in the pathogenesis of depression. UBTF can activate ribosomal RNA (rRNA) transcription in nucleoli (Sanij and Hannan, 2009; Pederson, 2011). Induction of rRNA and other nucleolar activities play a role in normal neural processes, such as neurite outgrowth and memory consolidation during spatial training (Capitano et al., 2016). As shown in the GO terminology, the target genes of UBTF are mainly enriched in this terminology (GO: 0070997 ∼ neuron death; GO: 1901990 ∼ regulation of mitotic cell cycle phase transition), including Wfs1 and Dtl. Wfs1 is required for normal endoplasmic reticulum function, while Wfs1-deficiency increases endoplasmic reticulum stress, impairs cell cycle progression, and triggers the apoptotic pathway, specifically in pancreatic beta-cells (Yamada et al., 2006). In response to ARS in Wfs1 CKO animals, downregulation of WNT7A in mPFC was observed (Yamada et al., 2006). WNT7A is a secreted signal transduction factor involved in axonal remodeling and synaptic differentiation (Ciani et al., 2011). These results suggest that Wfs1 may alleviate stress-induced depressive behaviors by regulating neuronal synaptic plasticity. Wfs1 and UBTF can participate in similar biological processes. Our results suggest that Wfs1 and UBTF may have the potential to be an anti-depressive drug target.

The miRNA is an endogenous non-coding RNA whose function is to degrade or inhibit the target gene’s translation process, thereby regulating gene expression at the post-transcriptional level. In this study, we observed nine miRNAs, that targeted three DEGs. It is expected that Wfs1 can adjust mmu-mir-17-5p, mmu-mir-466f-3p, mmu-mir-466m-3p, mmu-mir-466o-3p, mmu-mir-466i-3p, mmu-mir-362-3p, mmu-mir-329-3p, mmu-mir-1960, mmu-mir-128-3p, and mmu-mir-1186a, and Dtl can adjust mmu-mir-539-5p, mmu-mir-34b-5p, and mmu-mir-706. Screening by qRT-PCR revealed that mmu-mir-17-5p and mmu-mir-7b-5p were upregulated. Although there is no current evidence to prove their direct role in depression, the target gene Wfs1 of mmu-mir-17-5p is considered a candidate genetic marker related to the relief of depression (Ferrua et al., 2019). Therefore, Wfs1/UBTF/mmu-mir-17-5 may play an important role in the pathogenesis of depression caused by long-term stress. The molecular mechanism of Wfs1 and its related molecules participating in depression need to be further investigated. Further in-depth studies of their mechanisms would contribute to a more precise diagnosis and efficient treatment of depressive disorder.

## Conclusion

Our current study found that the cell cycle and the chemokine signaling pathway are important in stress-induced depression. Then, we identified Wfs1, which has significant relevance with the cell cycle biological processes, and screened out its related TF and miRNAs, including UBTF, mmu-mir-17-5p, and mmu-mir-7b-5p as potential experimental targets in the hippocampi of depressed mice. Further qRT-PCR results show that Wfs1 was down-regulated and UBTF, mmu-mir-17-5p, and mmu-mir-7b-5p were up-regulated in the CUMS group with healthy controls. In conclusion, Wfs1 and related molecules are key genes involved in depression. Further experiments are required to probe the functions of these genes in the pathogenesis of depression.

## Data Availability Statement

The datasets presented in this study can be found in online repositories. The names of the repository/repositories and accession number(s) can be found in the article/Supplementary Material.

## Ethics Statement

The animal study was reviewed and approved by the Zhejiang University.

## Author Contributions

JY and CC designed the experiments. LL performed the experiments. JY and XJ wrote the manuscript, analyzed the data, and collected the samples and delivered them. English has been professionally checked. All authors contributed to the article and approved the submitted version.

## Conflict of Interest

The authors declare that the research was conducted in the absence of any commercial or financial relationships that could be construed as a potential conflict of interest.

## Footnotes

1 https://www.ncbi.nlm.nih.gov/geo/

2 http://metascape.org/gp/#/main/step1

3 https://string-db.org/

4 http://www.networkanalyst.ca/faces/home.xhtml
