# Supplementary material for: Wfs1 and Related Molecules as Key Candidate Genes in the Hippocampus of Depression
Source: Front Genet. 2021 Jan 22;11:589370. doi: 10.3389/fgene.2020.589370 (PMC7863986; doi:10.3389/fgene.2020.589370)
Supplement: Supplementary file 1 [file Image_1.pdf]

A

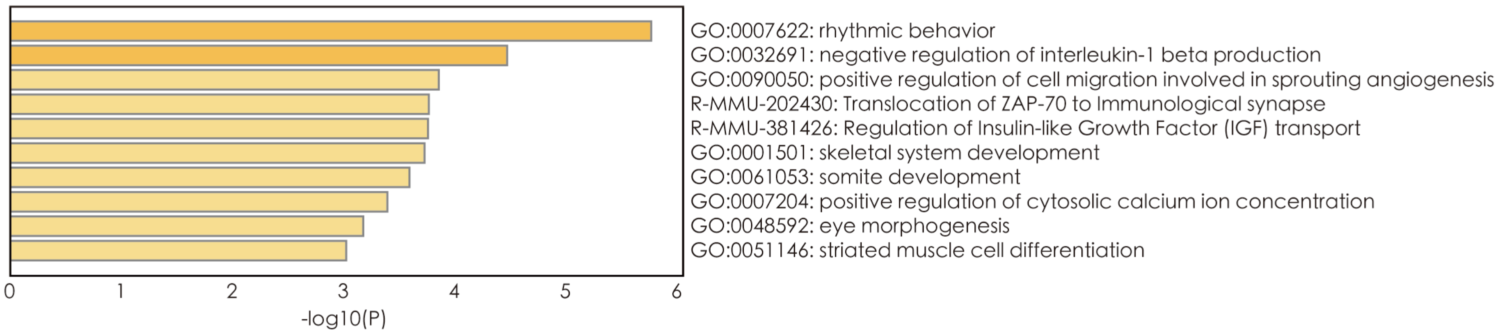
GO:0007622: rhythmic behavior
GO:0032691: negative regulation of interleukin-1 beta production
GO:0090050: positive regulation of cell migration involved in sprouting angiogenesis
R-MMU-202430: Translocation of ZAP-70 to Immunological synapse
R-MMU-381426: Regulation of Insulin-like Growth Factor (IGF) transport
GO:0001501: skeletal system development
GO:0061053: somite development
GO:0007204: positive regulation of cytosolic calcium ion concentration
GO:0048592: eye morphogenesis
GO:0051146: striated muscle cell differentiation
0
1
2
3
4
5
6
-log10(P)

B

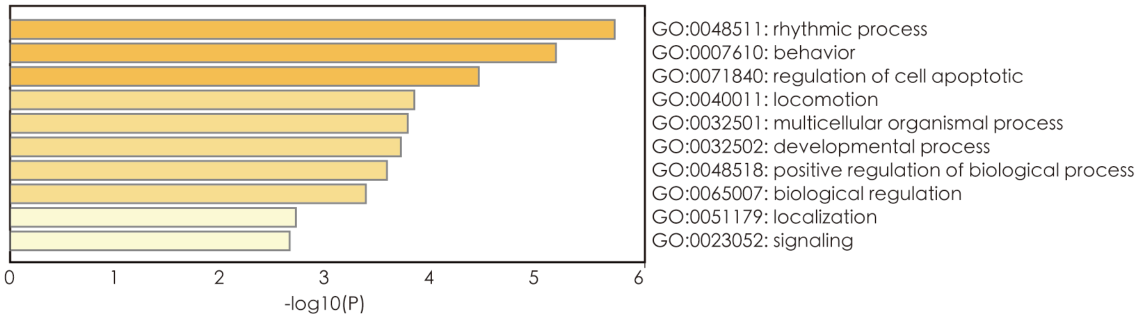
GO:0048511: rhythmic process
GO:0007610: behavior
GO:0071840: regulation of cell apoptotic
GO:0040011: locomotion
GO:0032501: multicellular organismal process
GO:0032502: developmental process
GO:0048518: positive regulation of biological process
GO:0065007: biological regulation
GO:0051179: localization
GO:0023052: signaling
0
1
2
3
4
5
6
-log10(P)
