# Supplementary figures and images for: Wfs1 and Related Molecules as Key Candidate Genes in the Hippocampus of Depression
Source: Front Genet. 2021 Jan 22;11:589370. doi: 10.3389/fgene.2020.589370 (PMC7863986; doi:10.3389/fgene.2020.589370)

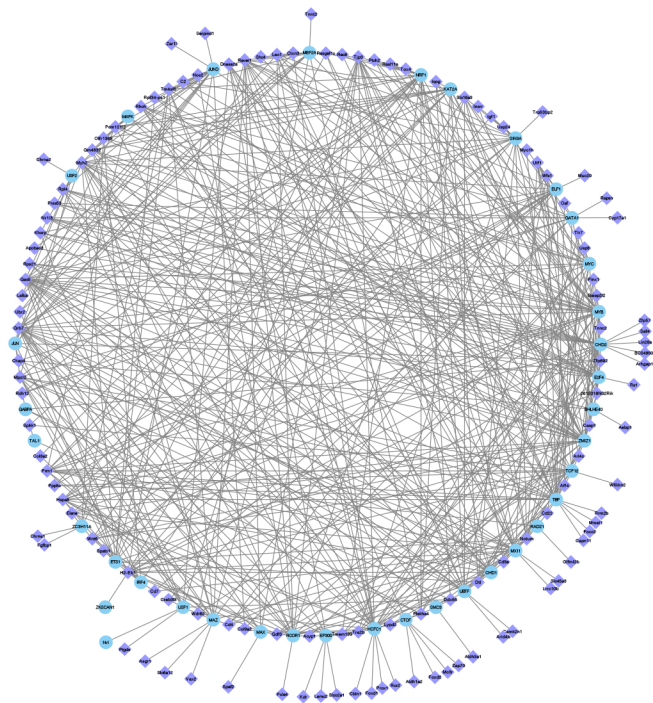

Supplement: Supplementary file 2 [file Image_2.pdf]

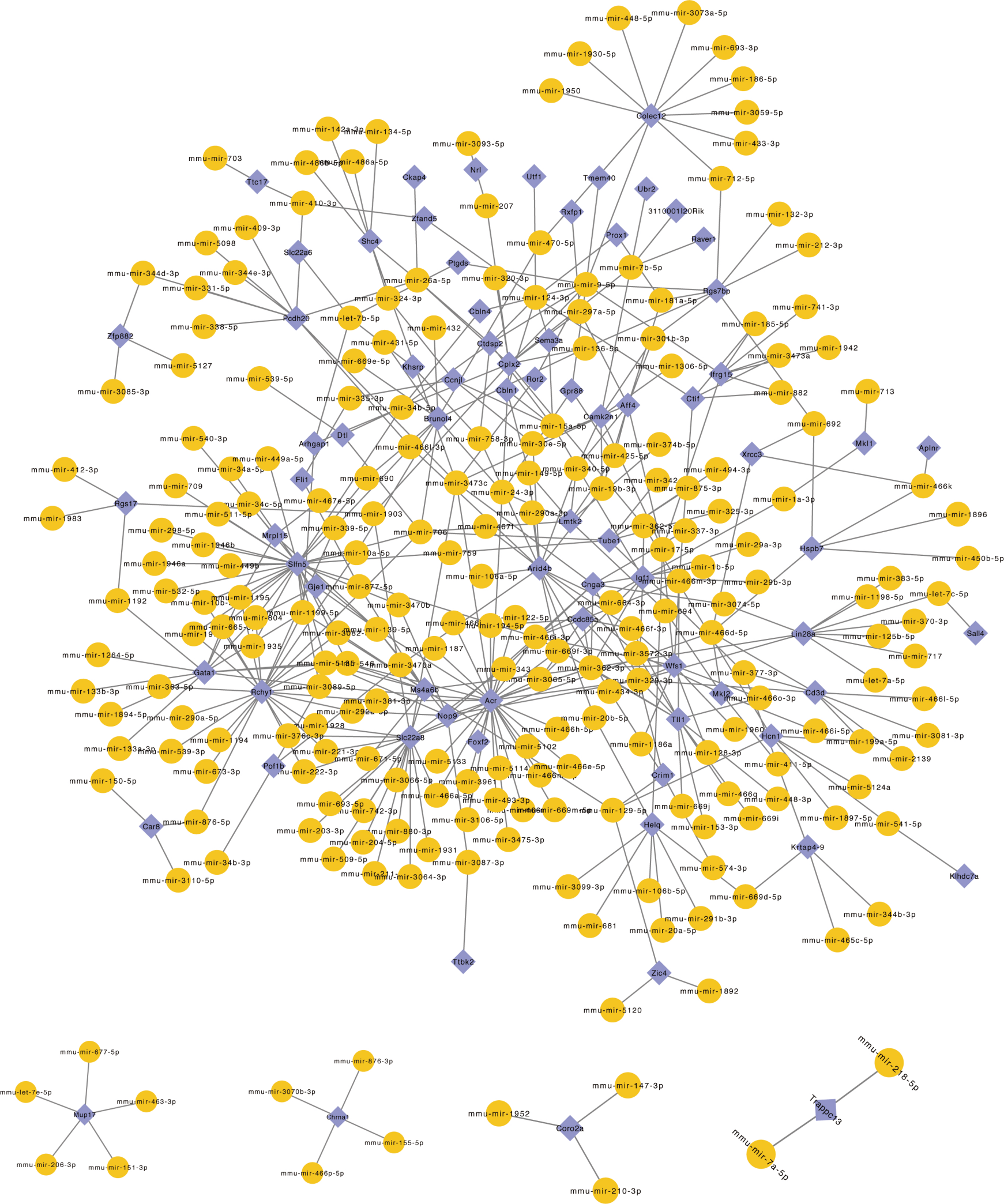

Supplement: Supplementary file 3 [file Image_3.pdf]
