# Supplementary material for: Wfs1 and Related Molecules as Key Candidate Genes in the Hippocampus of Depression
Source: Front Genet. 2021 Jan 22;11:589370. doi: 10.3389/fgene.2020.589370 (PMC7863986; doi:10.3389/fgene.2020.589370)
Supplement: Supplementary file 4 [file Table_1.docx]

**Supplementary Fig.1.** 325 DEGs enrichment analysis results obtained on Metascape website. (A)Select the first 10 biological processes and display them as a bar graph according to the enrichment score.(B)Select the first 10 KEGG pathway and display them as a bar graph according to the enrichment score.

**Supplementary Fig.2.** Distribution features of the expression situation after normalization of all samples. Network of TF‐DEG was obtained from ENCODE database. ENCODE, Encyclopedia of DNA Elements. A total of 529 associations between 36 TFs and 123 DEGs were found. As is shown, UBTF regulated 6 DEGs (Wfs1, Raver1, Dtl, Camk2n1, Arid4b, Aff4). ELF1 regulated 6 DEGs (Wfs1, Aff4, Utf1, Raver1, Ckap4). TBP regulated 4 DEGs (Wfs1, Aff4, Raver1, Dtl). MAZ regulated 3 DEGs (Wfs1, Aff4, Raver1, Dtl)

**Supplementary Fig. 3.** Network of miRNA‐DEG was obtained from TarBase and miRTarBase database. 5 large‐pairing pictures were generated . As a result, a total of 436 associations between 257 microRNAs and only 71 DEGs were found.
